# Human papillomavirus (HPV) genotyping and prognostic value of HPV E4 protein and transcription factors NANOG and SOX11 in atypical p16 patchy squamous epithelium of cervix

**DOI:** 10.2478/raon-2025-0038

**Published:** 2025-07-08

**Authors:** Maja Kebe Radulovic, Anja Ostrbenk, Mario Poljak, Margareta Strojan-Flezar

**Affiliations:** Institute of Pathology, Faculty of Medicine, University of Ljubljana, Ljubljana, Slovenia; Institute of Microbiology and Immunology, Faculty of Medicine, University of Ljubljana, Ljubljana, Slovenia

**Keywords:** p16, cervical intraepithelial neoplasia, genotype, human papillomavirus, NANOG, SOX11

## Abstract

**Background:**

Immunohistochemical staining for p16 is used to differentiate precancerous cervical lesions in tissue samples, but the interpretation of patchy p16 expression remains challenging. We performed human papillomavirus (HPV) genotyping and evaluated immunohistochemical expression of HPV E4 protein – a marker for transient infections, stem cell transcription factor NANOG, and transcription factor SOX11 to detect possible high-grade squamous lesions in atypical p16 patchy squamous epithelium.

**Materials and methods:**

We analyzed 24 cervical tissue samples with atypical squamous epithelium and patchy p16 expression along with the following controls: 11 cases of atypical squamous epithelium with null p16 expression, 9 condylomas, 12 cases of cervical intraepithelial neoplasia (CIN) grade 1, 11 cases of CIN2, and 9 cases of CIN3. In addition, HPV genotyping of tissue and related cervical smears from up to two years prior to biopsy was performed. Immunohistochemical staining for Ki67, HPV E4, NANOG, and SOX11 was performed and compared with follow-up data.

**Results:**

High-risk HPV infection was detected in 6/24 cases with patchy p16 expression and HPV E4 was expressed in 1/24 cases with patchy p16, weak NANOG expression was found in 11/24 cases with patchy p16 expression while no SOX11 expression was observed. During 10 months of follow-up, additional CIN1 and two CIN3 were identified, and another CIN1 and CIN3 after 5 and 6 years, accordingly.

**Conclusions:**

Our study showed that atypical squamous epithelium with patchy p16 expression poses a risk for highgrade precancerous lesions, harbouring high-risk HPV infection. Novel markers may hold diagnostic value in other specific contexts.

## Introduction

Histopathological diagnosis of precancerous lesions of the cervix is a prerequisite for treatment decisions for asymptomatic women who had cellular abnormalities detected in cervical smears (1). Following the publication of the Lower Anogenital Squamous Terminology Standardization Project for HPV-Associated Lesions (LAST) recommendations, a two-tiered classification system was established for the most common precancerous changes, namely low-grade squamous intraepithelial lesions (LSIL) and high-grade squamous intraepithelial lesions (HSIL), reflecting the association with human papillomaviruses (HPV) (2). LSIL is typically associated with productive and transient HPV infections that carry a low risk of progression, whereas HSIL is linked to a transforming and persistent HPV infections with a high risk of progression to cancer (3-6). In light microscopy evaluation of changes with intermediate histomorphological features, previously classified as cervical intraepithelial neoplasia grade 2 (CIN2), it is not always possible to reliably determine whether it is LSIL or HSIL; moreover the histomorphological appearance of CIN2 can overlap with various benign conditions (2,5).

To improve the diagnosis of CIN2, the LAST guidelines recommend immunohistochemical staining for cyclin-dependent kinase inhibitor p16 as a surrogate marker for transforming HPV infection (2). A strong diffuse immunohistochemical reaction en block for p16 is significantly associated with HSIL (2). In the foundational literature, positive p16 was not a mandatory criterion for the diagnosis of CIN2, although in longitudinal studies, CIN2 with negative p16 usually regressed (6-8).

Recent studies report significant increase in the use of p16 to define these lesions, while also observing a discrepancy between p16 results and histomorphological assessment in more than 30% of CIN2 cases (9-11). An additional challenge is ensuring the correct interpretation of patchy p16, which may not meet the aforementioned criteria but may be associated with HSIL in a fraction of women (9, 11-13).

Several studies have explored the prognostic value of p16 immunostaining in the follow-up of CIN2. In one study, 220 CIN2 cases were analysed and categorized by p16 expression into block-positive (n = 40), negative (n = 130), and ambiguous (n = 50) groups (11). During a 12-month follow-up, HSIL was detected in 14 of 40 (35%) block-positive cases, 2 of 130 (1.5%) p16-negative cases, and 8 of 50 (16%) ambiguous cases. The ambiguous group was further subclassified into strong/basal, strong/focal, and weak/diffuse patterns, all of which showed similar HPV detection rates (28–35%) and clinical outcomes. In the other studies with the follow-up periods of 12 and 36 months respectively, p16-negative CIN2 lesions consistently demonstrated a high likelihood of regression and no observed progression, while p16-positive CIN2 lesions had a significantly higher risk of progression to CIN3 (10-24%) (7,8).

Accurate histopathological diagnosis of cervical lesions prevents overtreatment, which can significantly compromise a woman’s fertility, particularly due to the premature birth, while insufficient treatment may be associated with progression to cervical cancer (14, 15).

The LAST guidelines also mention the use of the proliferation marker Ki67. It is only recommended for ambiguous or technically inadequate reactions with p16 and not routinely, as its sensitivity and specificity are lower compared to p16 (2, 16, 17).

Expression of the E4 protein of HPV has also been described in previous publications as a potential marker of productive HPV infection, both in the cervix and in skin lesions, anal, and oral mucosa (18-24). Studies have shown that it stains a larger proportion of CIN2 than CIN3 (25-28). In CIN3, combined lesions with productive and transforming infection predominated (28).

A new potential marker for dysplastic changes in squamous epithelium is NANOG, a transcription factor of embryonic stem cells (29). It is mostly not expressed in other normal human tissues in adults, except in the ovary and testis, but is frequently expressed in various types of carcinomas in the head and neck region, lungs, esophagus, stomach, colon, pancreas, liver, bladder, prostate, testicles, and ovaries (29-31). It is also present in precancerous changes of the squamous epithelium of the head and neck, cervix, and glandular epithelium of the stomach (29, 30).

NANOG expression in the cervix is primarily cytoplasmic, with weak positivity observed in some glandular cells of normal tissue, although some studies report its absence, including in the cervical transformation zone (30, 32). The findings in thus far limited studies vary; one study showed focal positivity in 30% of CIN1 cases but no positivity in CIN3 cases (32). In invasive squamous cell carcinoma, NANOG expression was heterogeneously positive in 23% of cases, with stromal cell positivity linked to disease progression (32). In another study, increased NANOG expression was found to correlate with the severity of dysplasia, peaking in invasive carcinoma (33). However, mRNA studies reported no significant differences between CIN2, CIN3, and invasive carcinoma, although expression was lower in negative controls, supported by immunohistochemistry (34, 35). In HPV16/18-positive cell cultures, NANOG enhances HPV long control region activity and elevates E6/E7 mRNA levels, while HPV E7 increases NANOG expression in epithelial cells. The NANOG binding sites are specific for high-risk HPV types (36, 37).

SOX11, a transcription factor involved in tumor development and immunosuppression, has been proposed as a marker for dysplastic changes in cervical squamous epithelium, with conflicting results (38). Some studies report significant expression in the basal cells of the normal cervix and in LSIL, while others find SOX11 expression exclusively in squamous cell carcinoma of the cervix, with no expression in normal cervical tissue (38, 39, 40).

The aim of this study was to evaluate the potential of the biomarkers HPV E4, NANOG and SOX11 together with HPV genotyping in atypical squamous epithelium with a patchy p16 expression to detect potential cervical precancerous lesions. The expression of biomarkers was compared to a control group consisting of atypical squamous epithelium with negative p16, condylomas, CIN1, CIN2 and CIN3 cases. Follow-up data on precancerous lesions were obtained from the National Cervical Cancer Screening Registry.

## Materials and methods

The study protocol was approved by the Medical Ethics Committee of Slovenia (Consent 0120107/2020/3) on March 17, 2020.

### Participants

Cervical tissue samples fixed in 10% buffered formalin and embedded in paraffin, from Institute of Pathology, Faculty of Medicine, University of Ljubljana (IP FM UL) archives were used for this retrospective study. Using the laboratory information system, we identified cases labeled “p16 neg” from gynecological biopsies between January 1, 2016, and December 31, 2018. The study focused on that period because it provided sufficient time for complete follow-up. Of the 320 matches, we excluded glandular changes, LSIL cases, and cases of cervical abrasion.

**FIGURE 1.**
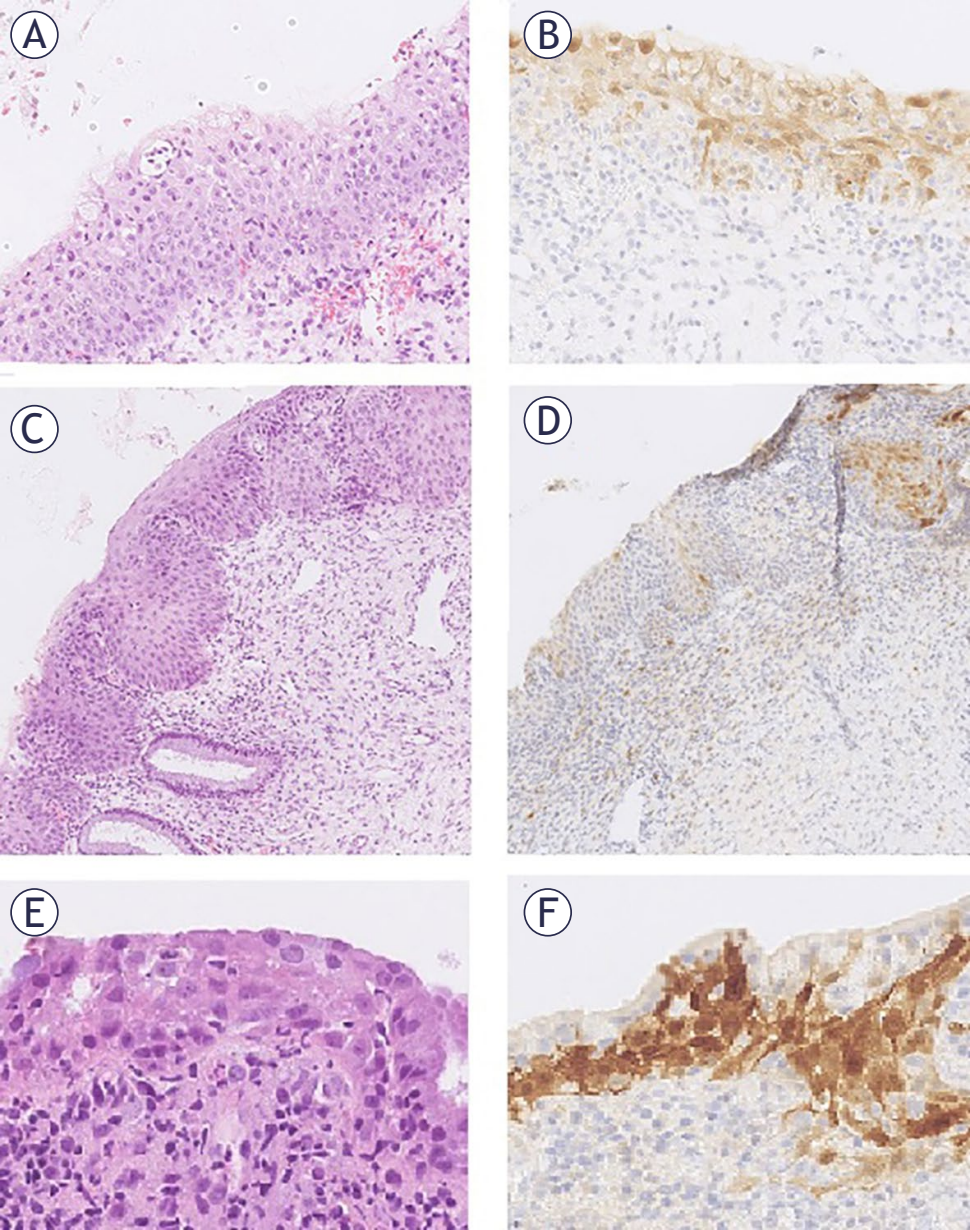
Images of atypical squamous epithelium cases with patchy p16 reaction: **(A–F)**. The same sample stained with HE and p16 at 200× magnification. Panels **(A), (C)**, and **(E)** show HE staining, while panels **(B), (D)**, and **(F)** show corresponding p16 staining.

We selected 20 unequivocally p16-negative i.e. p16 null atypical squamous epithelium cases and 56 with patchy p16 expression. After review, cases with sufficient tissue were retained, 11 p16-negative (null) cases and 24 with patchy nuclear and cytoplasmic positivity (Figure 1).

Atypical squamous epithelium was morphologically classified as such when the proliferating squamous or metaplastic epithelium exhibited nuclear atypia, characterized by enlarged nuclei and irregular nuclear membranes. Cytoplasmic differentiation was minimal or absent in the in the middle and superficial thirds of the epithelium.

Control group included 9 condylomas, 12 CIN1, 11 CIN2, and 9 CIN3, matched for age (±5 years) with atypical squamous epithelium cases with patchy p16 reaction, diagnosed at IP FM UL between January 1, 2015, and December 31, 2019. All slides in the study were independently reviewed by two pathologists. Eligible tissue samples were identified through our laboratory information system. After the review, samples with adequate material for additional testing were selected. The exclusion criteria specified that no lesions classified as higher grade than the defining group were present in the slides from these women.

The final study group, in which all immunohistochemical (IHC) stainings and HPV genotyping were performed, included 17 excision biopsies and 7 conizations in the group with patchy p16 reaction, 9 excisions and 2 conizations in the group with null p16 reaction, 6 excisions and 3 conizations in the group with condylomas, 5 excisions and 7 conizations in the CIN1 group, 5 excisions and 6 conizations in the CIN2 group and 2 excisions and 7 conizations in the CIN3 group.

### HPV Genotyping

HPV genotyping was performed at the Institute of Microbiology and Immunology (IMI) FM UL. The samples were prepared according to previously described in-house protocol (5). Briefly, we cleaned the microtome with xylene and a DNA decontamination solution before cutting 5 sections of 10 μm from the paraffin block, with first two sections being discarded. A negative control (leiomyoma tissue) was cut between consecutive samples. A new blade was used for each sample. The last tissue section was designated for HE to assess the presence of changes in the remaining tissue block. In addition, corresponding cervical smears of the same patients that were taken up to two years before the tissue biopsy were tested as well.

For the DNA isolation, commercially available QIAamp DNA Mini Kit (Qiagen, Hilden, Germany) was used and HPV genotyping was performed utilizing highly sensitive Allplex HPV28 Detection Kit (Seegene, Seoul, South Korea), both following the manufacturer’s instructions. Latter enables individual detection of 28 HPV genotypes: HPV6, 11, 16, 18, 26, 31, 33, 35, 39, 40, 42, 43, 44, 45, 51, 52, 53, 54, 56, 58, 59, 61, 66, 68, 69, 70, 73, 82. For further analysis, the latest IARC classification was followed, where the following 12 HPV genotypes were considered as high-risk: 16, 18, 31, 33, 35, 39, 45, 51, 52, 56, 58, and 59 (41) and the remaining 16 were categorized as low-risk (lrHPV) genotypes.

### Immunohistochemical Methods

Immunohistochemical analyses for p16, Ki67, E4 HPV, NANOG and SOX11 were conducted on 3-4 μm sections of formalin-fixed, paraffin-embedded tissue. We performed the immunohistochemical reactions which are regularly performed in routine practice without controls, namely Ki67 (monoclonal antibody, clone MIB-1, Dako, Agilent Technologies, Santa Clara, California, USA), p16 (antibody against p16INK4a protein) (clone E6H4, Ventana/Roche), SOX11 (Mouse monoclonal antibody, clone MRQ-58, Cell Marque, Rocklin, California, USA).

Immunohistochemical reactions for E4 HPV were performed using commercially available monoclonal antibodies against E4 (XR-E4-1) (IVD, Labo Bio-medical Products, Rijswijk, Netherlands), which react with the E4 protein from at least the following HPV strains: 6, 11, 16, 18, 27, 31, 33, 35, 39, 42, 43, 44, 45, 51, 52, 53, 56, 57, 58, 59, 66, 67, 70, and 74. Both negative and positive controls were included on each slide. For the negative control, we used leiomyoma tissue, and for the positive control, a case of CIN1.

Immunohistochemical reactions for NANOG were performed using commercially available monoclonal antibodies against NANOG (Cell Signaling, dilution 1:200) (Merck, Kenilworth, New Jersey, USA). One negative and three positive controls were included on each slide. For the negative controls, we used normal endocervical tissue and for the positive controls a non-keratinizing squamous cell carcinoma of the oropharynx, a seminoma of the testis, and CIN3..

The staining process for all immunohistochemical reactions was performed automatically using the BenchMark XT apparatus (Ventana, Tucson, Arizona, USA), with the ultraVIEW detection system and/or OptiVIEW DAB Detection Kit (Roche, Basel, Switzerland).

The criteria for the evaluation were adjusted to the different expressions of the markers. Ki-67 was evaluated as a positive reaction with nuclear staining. Parabasal reaction in the squamous epithelium was considered normal (0); otherwise, it was assessed by thirds of the thickness of squamous epithelium, namely 1 (predominantly lower 1/3), 2 (predominantly lower 2/3), 3 (full thickness) (42).For p16, a positive tissue reaction was assessed as diffuse, strong staining of cytoplasm and nuclei in at least the lower third of the epithelium (en block) (2). The reaction was evaluated with 3 levels: 0 for completely negative reaction, 1 for reaction with isolated stained nuclei and cytoplasm (patchy), 2 for positive reaction en block as described above. For HPVE4, a positive tissue reaction was assessed as cytoplasmic staining of at least one squamous epithelial cell (22, 27). For NANOG, a positive reaction was assessed as staining of the cytoplasm or nucleus (43, 44). The reaction was evaluated in three levels: 1 for weak reaction stronger than endocervical cells in the control, 2 for moderate reaction similar to CIN3, and 3 for strong reaction as seen in the nuclei of control seminoma cells. SOX11 was evaluated as a positive reaction with nuclear staining of epithelial cells (38, 39).

### Follow-up

Follow-up data on cytology, HPV testing and tissue biopsy diagnosis from the date of sample collection until May 30, 2024 were obtained from National Cervical Cancer Screening Registry ZORA (45).

### Statistical Analysis

Statistical analysis was conducted using IBM SPSS Statistics 27. The Spearman correlation test was applied due to the data’s non-parametric nature, with p < 0.05 as the significance threshold.

## Results

The age of the women included in the study ranged from 20 to 75 years (average 39 years). There was no statistical difference in ages between groups, except between the group with p16 null reaction and the condyloma group (r=-0.457, p=0.021), because women in the condyloma group were significantly younger (mean age 33 years) compared to the group with p16 null (mean age 42 years).

The results of immunohistochemical reactions for p16, Ki67, HPVE4, NANOG, and Sox11 with HPV genotyping in cervical tissue biopsies and cervical smears, together with follow-up data are presented in Tables 1-4.

**TABLE 1.** Immunohistochemical reactions to various biological markers and human papillomavirus (HPV) genotyping in tissue biopsy and previous cervical smear, where available, by study group

Group	p16	N	Ki67	N	E4 HPV	N	Nanog	N	HPV genotyping	N tissue	N smear
**p16 null N = 11**	Null	11	0	5	Neg.	11	Neg.	7	Neg	9	3
	Patchy	0	1/3	4	Pos.	0	1	4	lrHPV only	2	1
	En Block	0	2/3	0			2	0	hrHPV	0	3
			3/3	2			3	0			
**p16 patchy N = 24**	Null	0	0	12	Neg.	23	Neg.	13	Neg	10	2
	Patchy	24	1/3	5	Pos.	1	1	11	lrHPV only	6	2
	En Block	0	2/3	3			2	0	hrHPV	8	12
			3/3	4			3	0			
**Condyloma N = 9**	Null	0	0	0	Neg.	7	Neg.	0	Neg	0	1
	Patchy	9	1/3	2	Pos.	2	1	9	lrHPV only	6	2
	En Block	0	2/3	1			2	0	hrHPV	3	1
			3/3	6			3	0			
**CIN1 N = 12**	Null	0	0	2	Neg.	2	Neg.	0	Neg	0	0
	Patchy	10	1/3	2	Pos.	10	1	12	lrHPV only	6	1
	En Block	2	2/3	4			2	0	hrHPV	6	3
			3/3	4			3	0			
**CIN2 N = 11**	Null	0	0	0	Neg.	3	Neg.	0	Neg	1	0
	Patchy	0	1/3	0	Pos.	8	1	7	lrHPV only	2	0
	En Block	11	2/3	2			2	4	hrHPV	8	3
			3/3	9			3	0			
**CIN3 N = 9**	Null	0	0	0	Neg.	7	Neg.	1	Neg	0	0
	Patchy	0	1/3	0	Pos.	2	1	3	lrHPV only	0	0
	En Block	9	2/3	0			2	3	hrHPV	9	4
			3/3	9			3	2			

1 0 = null or parabasal reaction of Ki67; 1/3 = Ki67 positive in predominantly lower 1/3 of epithelium; 2/3 = Ki67 positive in predominantly lower 2/3 of epithelium; 3/3 = Ki67 positive in full thickness of epithelium; CIN = cervical intraepithelial neoplasia; hrHPV = high risk HPV; lrHPV = low risk HPV; Neg. = negative reaction; Pos. = positive reaction

**TABLE 2.** Follow-up histology, cytology, human papillomavirus (HPV) result by study groups (p16 null and p16 patchy)

Group	Follow up histo	N	Follow up cyto	N	Follow up HPV	N
**p16 null (11 cases, 9 biopsies)**	Neg	0	Neg	5	Neg	5
	CIN1	1	ASC-US	4	Pos	3
	CIN2	1	LSIL	0		
	CIN3	1	HSIL	0		
**p16 patchy (24 cases, 17 biopsies)**	Neg	0	Neg	5	Neg	4
	CIN1	2	ASC-US	6	Pos	5
	CIN2	0	LSIL	4		
	CIN3	3	HSIL	2		

1 ASC-H = atypical squamous cells, cannot exclude HSIL; ASC-US = atypical squamous cells of undetermined significance; CIN = cervical intraepithelial neoplasia; Follow up cyto = cervical smears from follow-up; Follow up histo = histological samples from follow-up; Follow-up HPV = HPV results using the Hybrid Capture II assay; HSIL = highgrade squamous intraepithelial lesions; LSIL = low-grade squamous intraepithelial lesions; Neg = negative; Pos = positive

1 Follow-up after radical resections (conizations, cervical amputations, hysterectomies) was not informative, as the lesion was completely removed; therefore, this data is not included. In the case of multiple consecutive examinations (cytology, HPV testing), we only considered the most pathological cytology results or the positive HPV test.

**TABLE 3.** Biomarkers in the primary biopsy and follow-up in the groups with atypical squamous epithelium with a patchy and a null reaction to p16

Group	Case	Ki67	E4 HPV	Nanog	HPV genotypes	Follow-up sample	Follow-up diagnosis	Time from primary biopsy to follow-up sample
**p16**	1	1/3	-	1+	31, 52	biopsy	CIN1	2 years
**null**	2	3/3	-	-	53	biopsy	CIN2	2 years
	3	1/3	-	-	-	biopsy	CIN3	4 months
**p16 patchy**	4	0	-	-	39	cone	CIN1	8 months
	5	1/3	-	-	-	cone	CIN1	6 years
	6	3/3	-	1+	53	cone	CIN3	5 years
	7	3/3	-	-	52, 53, 56, 66, 73	Cervical amputation	CIN3	10 months
	8	1/3	-	-	44	cone	CIN3	10 months

1 0 = null or parabasal reaction of Ki67; 1/3 = Ki67 positive in predominantly lower 1/3 of epithelium; 2/3 = Ki67 positive in predominantly lower 2/3 of epithelium; 3/3 = Ki67 positive in full thickness of epithelium; - = negative reaction, + = positive reaction; CIN = cervical intraepithelial neoplasia; HPV = human papillomavirus

**TABLE 4.** Comparison of different biological markers between groups using Spearman correlation

Groups	p16	Ki67	HPV E4	Nanog	lrHPV tissue	hrHPV tissue	hrHPV: tissue+smear	lrHPV: tissue+smear
p16 null vs. p16 patchy	**r = 1 p = 0.000**	r **=** 0.000	r **=** 0.116	r **=** 0.089	r **=** 0.209	**r = 0.384 p = 0.025**	r **=** 0.274	r **=** 0.285
p16 patchy vs. condyloma	r = 0.000	**r = 0.535 p = 0.001**	r =0.280	**r = 0.494 p = 0.004**	**r = 0.458 p = 0.007**	r = 0.000	r=-0.186	**r = 0.494 p = 0.004**
p16 patchy vs. CIN1	**r = 0343 p = 0.041**	**r = 0.35 p = 0.037**	**r = 0.81 p = 0.000**	**r = 0.532 p = 0.001**	**r = 0.354 p = 0.034**	r = 0.161	r = -0.039	**r = 0.359 p = 0.032**
p16 patchy vs. CIN2	**r = 1 p = 0.000**	**r = 0.66 p = 0.000**	**r = 0.728 p = 0.000**	**r = 0.632 p = 0.000**	r = -0.193	**r = 0.45 p = 0.007**	r = 0.176	r = -0.089
p16 patchy vs. CIN3	**r = 1 p = 0.000**	**r = 0.683 p = 0.000**	r = 0.280	**r = 0.575 p = 0.000**	**r = -0.375 p = 0.032**	**r = 0.594 p = 0.000**	**r = 0.433 p = 0.012**	**r = -0.433 p = 0.012**
p16 null vs. condyloma	**r = 1**	**r = 0.617** p = 0.004	r = 0.369	**r** = **0.664** p = 0.001	**r** = **0.704** p = 0.001	**r** = **0.464** p = 0.039	r = 0.066	**r** = **0.818** p = 0.000
p16 null vs. CIN1	**r = 0.963 p = 0.000**	r = 0.394	**r = 0.84 p = 0.000**	**r** = **0.691 p** = **0.000**	**r** = **0.568 p** = **0.005**	**r** = **0.569 p** = **0.005**	r = 0.233	**r** = **0.652 p** = **0.001**
p16 null vs. CIN2	**r = 1**	**r = 0.735 p = 0.000**	**r = 0.756 p = 0.000**	**r** = **0.728 p** = **0.000**	r = 0.000	**r** = **0.832 p** = **0.000**	**r** = **0.455 p** = **0.034**	r = 0.204
p16 null vs. CIN3	**r** = **1**	**r = 0.783** p = 0.000	r = 0.369	**r** = **0.665** p = 0.001	r = -0.302	**r** = **1**	**r** = **0.739** p = 0.000	r = -0.302
Atypia (p16 patchy and null) vs. CIN2	**r = 0.811** p = 0.000	**r = 0.607** p = 0.000	**r = 0.751** p = 0.000	**r** = **0.598** p = 0.000	r = -0.125	**r** = **0.521** p = 0.000	r = 0.231	r = -0.007

1 **Bold font** = statistically significant correlation; CIN = cervical intraepithelial neoplasia; hrHPV: tissue = high risk HPV detected in tissue; hrHPV: tissue+smear = high risk HPV detected in tissue and in cervical smear taken before biopsy; **HPV =** human papillomavirus; lrHPV: tissue = low risk HPV detected in tissue; lrHPV: tissue+smear = low risk HPV detected in tissue and in cervical smear taken before biopsy; p = p value; p16 null = atypical squamous epithelium with a null p16 expression; p16 patchy = atypical squamous epithelium with a patchy p16 expression; r = correlation coefficient

1 Colour scale based on correlation coefficient value: **Table:** 

1	
0,9	
0,8	
0,7	
0,6	
0,5	
0,4	
0,3	
0,2	
0,1	
0	
-0,1	
-0,2	
-0,3	
-0,4	
-0,5	
-0,6	
-0,7	
-0,8	
-0,9	
-1

### HPV genotyping

For all cases, a moderate correlation between HPV genotypes detected in cervical smears and in histological sample was observed, with a special emphasis that cervical smears were taken up to 2 years prior to tissue biopsy samples (r=0.526, p=0.001). The correlation was statistically significant for hrHPVs (r=0.633, p=0.000) but not significant for lrHPVs (r=0.261, p=0.114).

In squamous epithelial atypia with null p16 expression the following HPVs were detected in tissue as a single infection: 53, 82.

In squamous epithelial atypia with patchy p16 expression the following HPVs were detected in tissue as either single/co-infection: 6, 16, 31, 39, 44, 52, 53, 56, 59, 66, 68, 58, 73.

In condylomas the following HPVs were detected in tissue as either single/co-infection: 6, 11, 16, 18, 44, 51.

In CIN1, the following HPVs were detected in tissue as either single/co-infection: 6, 16, 31, 40, 51, 52, 53, 56, 61, 66, 68, 70.

In CIN2, the following HPVs were detected in tissue as single infections: 16, 31, 58, 73, while coinfections included 18 and 58, 26 and 53, and 58 and 59.

In CIN3, the following HPVs were detected in tissue as single infections: 16, 18, 31, 51, while coinfections included 16 and 52.

Regarding various hrHPVs, HPV16, either single or as a coinfection, was present in none of the cases from the group with a completely negative reaction to p16, in 1 case from the group with a patchy reaction to p16, in 2 cases in the CIN1 group and the condyloma group, in 2 cases in the CIN2 group, and in 4 cases in the CIN3 group.

### E4 HPV

E4 HPV was most frequently expressed in thickened epithelium with hyper- and parakeratosis of CIN1 and CIN2 than in other groups, including majority of atypical squamous epithelia with patchy or null p16 expression.

E4 HPV was negative in all cases with negative tissue HPV genotyping and all 6 cases with confirmed single HPV6 infection, including five condyloma cases, one CIN1 case and one case in the patchy p16 group.

**FIGURE 2.**
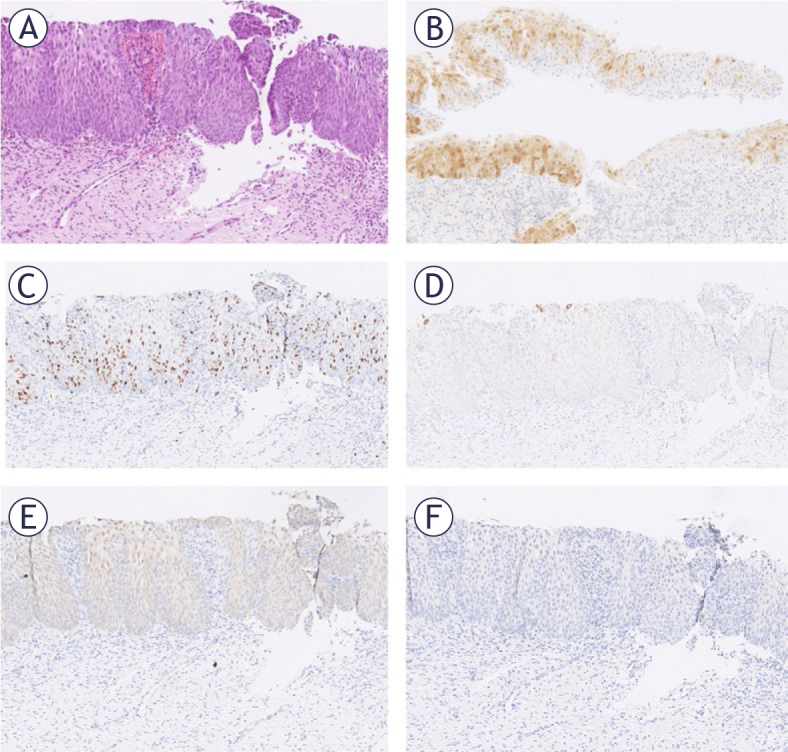
Only case in the group with atypical squamous epithelium with patchy p16 expression and positive reaction for E4 human papillomavirus (HPV). The tissue was positive for HPV16 and 31 and the follow-up was negative. The same section at 100x magnification: **(A)** HE staining; **(B)** p16 staining, **(C)** Ki67 staining, **(D)** E4 HPV staining, **(E)** Nanog staining, F. SOX11 staining.

An exception was a case with positive E4 HPV in the atypical squamous epithelium with patchy p16 expression group and with HPV16 and 31, where NANOG was 1+ and Ki67 was expressed throughout the epithelium (Figure 2).

**FIGURE 3.**
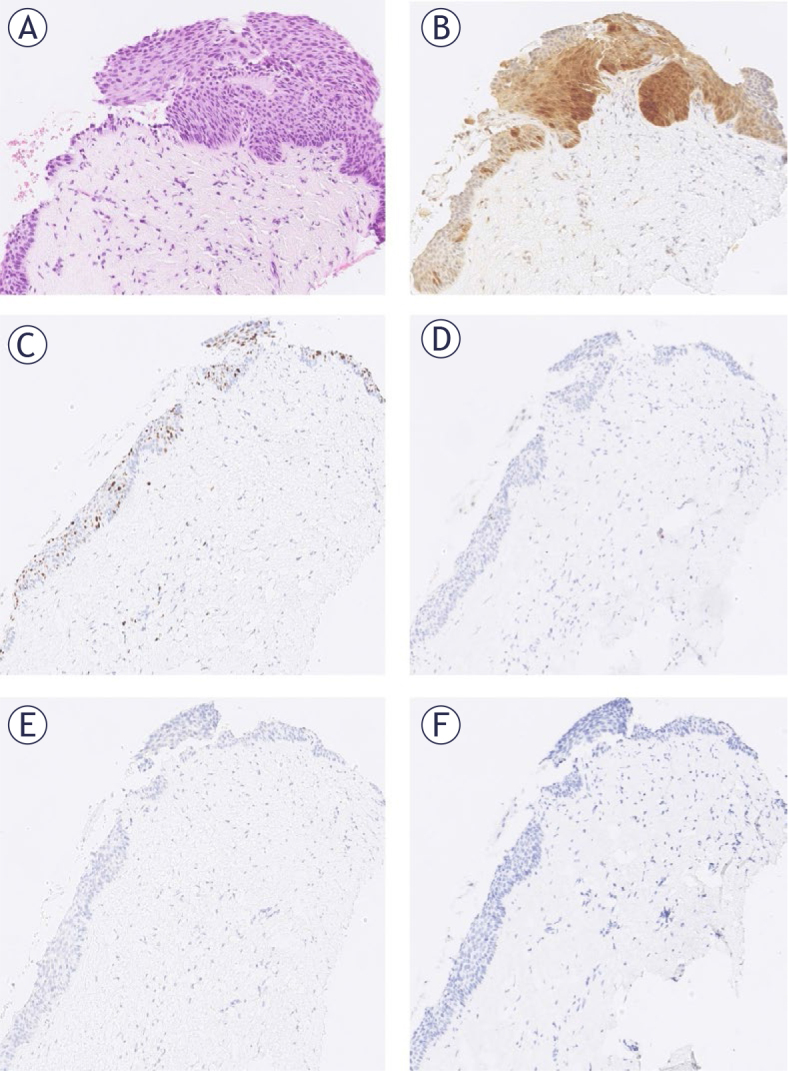
One of the three cases in the group with atypical squamous epithelium with patchy p16 with CIN3 on follow-up. The tissue was positive for human papillomavirus (HPV) 52, 53,56, 66, 73. The same section at 100x magnification: **(A)** HE. **(B)** p16 staining. **(C)** Ki67 staining. **(D)** E4 HPV staining. (**E**) Nanog staining. (**F**) SOX11 staining.

Another exception were two cases of negative E4 HPV in the CIN1 group were, namely one conization case with HPV16 detected on genotyping and in another biopsy case with HPV6 infection, where NANOG was 2+ and Ki67 was expressed throughout the epithelium.

In the CIN2 group, three cases were negative for E4 HPV. One was a biopsy positive for HPV73, another was a conization case with negative HPV genotyping, and the third was a conization case with HPV16 infection. All three cases had weak NANOG expression (1+).

In the CIN3 group, E4 HPV was positive in two cases. One case involved a combined (CIN2 and CIN3) lesion with surface koilocytic changes and HPV16, while the other had no koilocytic changes but was positive for HPV31.

### NANOG

The reaction to NANOG was predominantly cytoplasmic, perinuclear, and occasionally nuclear. There was a weak (1+) positive reaction to NANOG in morphologically normal squamous epithelium outside the lesion. Normal glandular epithelium was negative for NANOG, as well as the normal metaplastic squamous epithelium. Atypical squamous epithelium with patchy p16 expression showed 1+ NANOG expression in 11 out of 24 cases, while the remaining cases were negative. Among the 11 NANOG-positive cases, 2 were HPV-negative. In contrast, among the 13 NANOG-negative cases, 7 were HPV-negative.

In a minority of CIN cases, NANOG expression was uneven, sometimes present only in the lower layers of dysplastic epithelium.

Considering a reaction of 2+ or greater (strong reaction), NANOG was positive in 9/20 cases of HSIL, in 1/12 cases of CIN1 (the one with HPV16 infection), but no strong reaction was observed in cases with p16 patchy/null atypical squamous epithelium.

### SOX11

**FIGURE 4.**
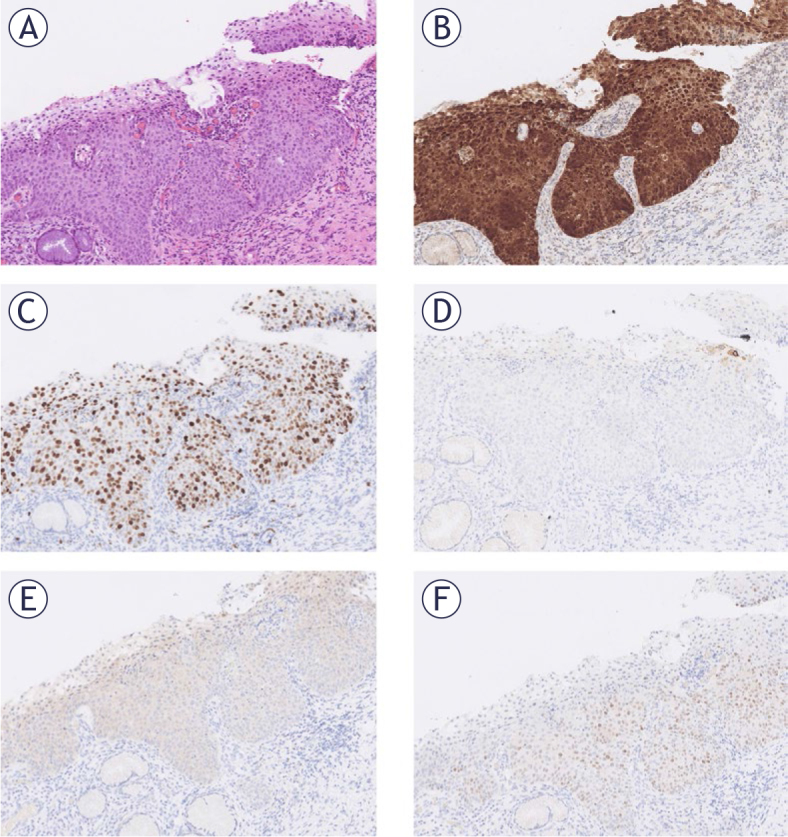
Immunohistochemical reactions in the only cervical intraepithelial neoplasia 3 (CIN3) case with a positive SOX11 reaction in study. The same section at 100x magnification: **(A)** HE. **(B)** p16 staining. **(C)** Ki67 staining. **(D)** E4 HPV staining. **(E)** Nanog staining. **(F)** SOX11 staining.

The immunohistochemical marker SOX11 was not included in the tables because it was negative everywhere except in one case. The only positive reaction for SOX11 was nuclear, in the basal part of CIN3 with HPV16 infection (Figure 4).

## Discussion

### HPV genotyping

Our study showed that atypical squamous epithelium with patchy p16 expression, which should be considered negative for HSIL according to the LAST guidelines, do not exclude high-risk HPV infection or HSIL in the follow-up, which is consistent with finding of previous studies (11, 23, 46). In particular, one third of cases with atypical squamous epithelium with patchy p16 expression had hrHPV, one forth had lrHPV, comperable to previous studies (11).

Our study group consisting of cases with atypical squamous epithelium with patchy p16 expression closely resembles CIN1 and condylomas in p16 expression, with no statistically significant difference in hrHPV positivity between them (Table 4). Notably, lrHPV are significantly more prevalent in CIN1 and condylomas than in atypical squamous epithelium with patchy p16 expression. In comparison, CIN2 had statistically more hrHPV and less lrHPV than atypical squamous epithelium with patchy p16 expression group. Conversely, there is a statistically significant difference between groups of atypical squamous epithelium with patchy p16 compared to atypical squamous epithelium with null expression. The latter contained no hrHPV, indicating a much lower risk for HSIL, in line with other studies (11).

### p16

We observed that patchy p16 staining, besides in the group with atypical squamous epithelium and patchy p16 expression, is frequently seen in LSIL with lrHPV infections. As expected, en bloc p16 staining appeared exclusively in HSIL and two CIN1 cases containing hrHPV (2). The only exception with positive p16 and negative HPV genotyping in tissue was a CIN2 case with initially positive high-risk HPV genotyping in cervical smear a month before our biopsy. The literature suggests that “HPV-negative” CIN may result from false negatives due to rare or latent infections, p53-related oncogenesis, CIN2 regression, or reactive changes mimicking CIN2, beside the technical issues (loss of tissue) (47, 48).

Our study confirmed that p16 strongly correlates with hrHPV infection, as no cases of completely p16-negative atypia tested positive for hrHPV. Clinically, this suggests that such patients are unlikely to develop high-risk precancerous changes. Notably, none of the CIN1 cases in our study exhibited a completely negative p16 reaction, which aligns with findings from other studies suggesting that p16 is not a surrogate marker for just transformative infection (2, 23, 49).

### Ki67

In the group with atypical squamous epithelium and patchy p16 expression, Ki67 showed fullthickness positivity in two of three cases that progressed to CIN3 during follow-up, making it the most reliable predictive marker for future HSIL in this group, consistent with the literature (8, 50). However, Ki67 alone would not be sufficient for risk stratification, as there is no clear distinction in its expression between groups with atypical squamous epithelium with patchy versus null p16 expression.

### E4 HPV

E4 HPV expression provided the clearest distinction between atypical squamous epithelium with patchy p16 and CIN1/2 groups, being significantly more frequent in CIN1/2 (Table 4). That is in line with another study, where E4 positivity increased with positivity of p16 reaction when p16 expression was limited to the lower two third of the epithelium, since two cases from CIN1 group were p16 positive (Table 1) (23). In the CIN3 group, E4 HPV was positive in one conventional CIN3 case and another with combined lesions (23, 50).

As a potential marker of productive HPV infection, E4 HPV was detected in only one case of squamous condyloma. This may not indicate a lack of productive HPV infection but rather that immunohistochemical staining for E4 HPV does not detect lesional cells related to HPV6, a common cause of condylomas (26). Notably, recent literature and manufacturer specifications no longer mention that this staining is unvalidated for HPV6, suggesting a need for reevaluation (21). Among other unvalidated HPV types in single infections, immunohistochemical staining reacted with HPV68-related lesion (one CIN2 case) but not with HPV73 (one CIN2 case). This selective reaction may complicate the clinical management E4 HPV negative CIN2, as an undetected lrHPV73 along with an en block p16 reaction could be mistaken for a transforming infection, leading to overtreatment.

### NANOG

NANOG expression was less frequently observed in atypical squamous epithelium with null or patchy p16 expression compared to normal squamous epithelium, where it typically exhibited a weak 1+ staining pattern. Notably, squamous metaplastic and glandular epithelium demonstrated a completely negative reactions to NANOG, consistent with findings from previous studies (30, 32). This observation supports the final histopathological diagnoses of immature or reactive squamous metaplasia in these lesions at least at some cases with patchy p16 staining.

No strong (2+) NANOG reaction was observed in the group with atypical squamous epithelium and was present in only one case within the LSIL group. This suggests a lower malignant potential in these lesions compared to HSIL. Similar findings have been reported in laryngeal dysplasia, where a strong cytoplasmic NANOG reaction was identified as an independent predictor of carcinoma, whereas weak reactions were not (29). Additionally, the same study noted “negligible” NANOG staining in normal laryngeal squamous epithelium (29). Potential explanations for the negligible NANOG expression in laryngeal epithelium compared to the consistently weak reactions observed in cervical epithelium include selective NANOG binding to high-risk HPV, though this does not fully explain its presence in not HPV related laryngeal dysplasia (36). We hypothesize that the absence of NANOG expression in squamous metaplastic and glandular epithelium may be just a characteristic of the original glandular epithelium, from which metaplastic squamous epithelium arises.

### SOX11

In our study, SOX11 expression in basal cells of the normal cervix, atypical squamous epithelium and LSIL was null, contrary to the literature (38). SOX11 was expressed in one case of CIN3 (Figure 4). Previous studies reported increased SOX11 expression in cervical squamous cell carcinoma and adenocarcinoma (40). Despite deeper tissue sectioning, no local invasion was observed in our case, leaving SOX11 expression in the group of HSIL.

### Follow-up

During follow-up, three incident cases of CIN3 were identified in the group of atypical squamous epithelium with patchy p16 expression in the initial biopsy. None of these three cases had expressed E4 HPV. All three had confirmed HPV infections, two with lrHPV and one with hrHPV. In contrast, one case in the group of atypical squamous epithelium with patchy p16 expression did express E4 HPV and had a hrHPV, and no precancerous changes were found upon further monitoring (Figure 2). These results support the hypothesis that E4 HPV may be an indicator of productive infection that could regress in the group with patchy p16 expression.

The main limitation of our study is small sample size. Thus, caution is needed in interpreting the results of our study. This underscores the need for additional studies with larger sample size, some of which are already in progress (25).”

## Conclusion

Atypical squamous epithelium with patchy p16 expression, which is considered negative for HSIL according to the LAST guidelines, do not rule out the presence of high-risk HPV infection or the possible development of HSIL in the follow-up. None of the CIN3 cases from atypical squamous epithelium with patchy p16 expression group identified during follow-up exhibited E4 HPV expression in the initial biopsy. All three cases had confirmed HPV infections—two with lrHPV and one hrHPV. In contrast, one case in this group that expressed E4 HPV harbored hrHPV but showed no precancerous changes upon further monitoring.

None of the cases with atypical squamous epithelium and patchy p16 expression exhibited strong NANOG reactivity, which was frequently observed in HSIL. Conversely, many cases in this group showed a completely negative NANOG reaction, similar to that seen in normal epithelium with squamous metaplasia.

No hrHPV genotypes were detected in a group of atypical squamous epithelium with null p16 expression, indicating a much lower risk for HSIL. However, this distinction between atypical squamous epithelium with patchy p16 expression and null p16 expression is not yet reflected in clinical guidelines. According to the LAST recommendations, two diagnostic options with different clinical paths exist for atypical squamous epithelium with negative p16 expression. First is that p16-negative HSIL should be interpreted as negative or not associated with HPV pathology, and second that a p16-negative CIN2 should be classified as LSIL. It is possible that patchy p16 expression indicates a tendency toward LSIL, while null p16 expression may suggest a negative result. However, additional test, such as HPV genotyping in the tissue, might be helpful.

Based on our results, we hypothesize that novel markers may hold diagnostic value in specific contexts: E4 HPV for identifying productive HPV infections in CIN1/2, null NANOG expression in atypical squamous epithelium belonging to squamous metaplasia, weak NANOG expression in normal squamous epithelium, strong NANOG expression in high-grade dysplasia and SOX11 for high-grade lesions progressing toward carcinoma. Further studies including larger number of well characterized samples are needed to confirm our findings.
